# Is Bocourt’s Terrific Skink Really So Terrific? Trophic Myth and Reality

**DOI:** 10.1371/journal.pone.0078638

**Published:** 2013-10-25

**Authors:** Stéphane Caut, Magaly Holden, Michael J. Jowers, Renaud Boistel, Ivan Ineich

**Affiliations:** 1 Estación Biológica de Doñana, Consejo Superior de Investigaciones Científicas (CSIC), Sevilla, Spain; 2 Centre d’Écologie Fonctionnelle et Évolutive (UMR CNRS 5175). École Pratique des Hautes Études, Biogéographie et Écologie des Vertébrés. Campus CNRS, Montpellier, France; 3 Institut de Paléoprimatologie, Paléontologie Humaine: Evolution et Paléoenvironnements, CNRS-INEE, Université de Poitiers, Poitiers, France; 4 Muséum National d'Histoire Naturelle, Département Systématique et Évolution, UMR CNRS 7205 (Origine, Structure et Évolution de la Biodiversité), Paris, France; Scottish Association for Marine Science, United Kingdom

## Abstract

Many scientists argue that our planet is undergoing a mass extinction event that is largely due to human influences. In this context, rediscoveries of species presumed to be extinct are encouraging and of great potential interest. During a 2003 expedition to New Caledonia, Bocourt’s terrific skink, *Phoboscincus bocourti*, was unexpectedly rediscovered on a small islet by one of us. This skink species had been described from a single specimen collected around 1872 in New Caledonia. Since that time, however, no data on the species’ biology, trophic interactions, or role in the ecosystem have been collected, making it difficult to follow the established conservation plan. In this study, we used a multidisciplinary approach involving natural history, anatomy, morphology, genetics, and stable isotopes to elucidate the ecology of Bocourt’s terrific skink. Over the course of three different expeditions to the islet (total of 55 days across 2005 and 2012), we captured 4 individuals and observed another 4 individuals. The species’ dentition and trophic ecology suggest that it is a top predator in its ecosystem and a major consumer of small terrestrial reptiles. Its high degree of genetic relatedness to another New Caledonian skink, which has a broad distribution, suggests that *P. bocourti* underwent genetic isolation at a geographical remote location, where dispersal or colonization was highly improbable. Moreover, the lack of genetic variation among the four individuals we captured may imply that a unique lineage, characterized by few inter-island exchanges, exists on the islet. Bocourt’s terrific skink may be the largest terrestrial squamate predator alive in New Caledonia today. As a result, it is likely vulnerable to habitat modifications and especially the invasive rodents found on this islet. Further information is necessary to assess the conservation plans and practices in place as no concrete changes have been made since the species’ rediscovery almost 10 years ago.

## Introduction

“There is no doubt that the terrific skink ruled over the smaller reptiles of New Caledonia as effectively as *Tyrannosaurus rex* did over the lesser dinosaurs of its era, or the lion does on Africa’s Serengeti today.” ([1]; speculation made ten years before *Phoboscincus bocourti*’s rediscovery)

While the human population continues to increase exponentially, 30% of amphibians, 12% of birds, and 21% of mammals are either threatened or already extinct [2]. Many scientists argue that our planet is undergoing a mass higher extinction event, mostly due to anthropogenic factors such as the overexploitation of natural resources, habitat fragmentation, urbanization, the wildlife trade, the introduction of invasive species, pollution, and/or global warning [3-6]. Reptiles seem to be especially vulnerable. A recent study conducted by over 200 world-renowned experts assessed the extinction risk of 1,500 randomly selected reptiles from across the globe; it revealed that almost one in five reptiles is struggling to survive [7]. It is thus not surprising that at least 19% of the world’s reptiles are threatened and in danger of extinction.

Rediscoveries of species that are presumed to be extinct are encouraging and generate both great interest and concern for conservationists and biogeographers [8-11]. Over the past 122 years, at least 351 species have been rediscovered, a number that continues to increase over time. In most cases, the rediscovery represents the first opportunity to collect data on the species since it was first described [12]. For example, many species have been described from just one or a few museum specimens that were collected decades or even centuries earlier. For instance, the snake species *Eirenis africana*, identified from a single museum specimen described in 1914, was rediscovered in Djibouti in 1999 [13], and the crested gecko of New Caledonia, *Correlophus ciliatus*, considered to be extinct for more than a century, was rediscovered in 1994 [14]. Once their habitats are better characterized, some species are more commonly encountered and found to be no more threatened than other species in the same genus.

The rediscovery of an extinct species may present a variety of regulatory challenges and raise the question of the direction future conservation efforts should take [15]. Although species rediscovery can draw media attention, promote conservation efforts, and encourage research aimed at understanding population declines [9,16], it may also spur unsupported optimism for the survival of the species [12]. Many rediscovered species remain seriously threatened with extinction and could go extinct if concerted efforts are not directed to their conservation [12]. Thus, the rediscovery of a species is the first of many steps, especially if the species’ ecology is poorly characterized or unknown.

The lizard fauna of New Caledonia (~120 species) is among the most diverse of the South Pacific; it is probably the richest in the world if land surface area is taken into account. Moreover, it is characterized by an exceptionally high level of endemism at both the genus (~75%) and species (~95%) levels [17,18]. The high speciation rate of New Caledonian lizard fauna is most likely a consequence of restricted species distributions: fine-scale microendemism is associated with local geological features that generate unique habitats [18]. 

Before the arrival of humans in New Caledonia, the archipelago was dominated by the gigantic, herbivorous *Meiolania* horned tortoise [1,19] and two top predators: a goanna, similar to Australia’s varanids [1,20], and an ancient member of an extinct pygmy crocodile group discovered in 1980 (*Mekosuchus inexpectatus*; see [20,21]) and that probably originated in Australia. Unfortunately, only two moderately-sized groups of lizards survived in New Caledonia: the Gekkota, which are represented by the regionally endemic Diplodactylidae and the more widely distributed Gekkonidae, and the Scincidae or the skinks (however, it should be noted that some authors suggest Scincidae is not a single family, but rather several). Among the skinks, the gigantic skink (*Phoboscincus bocourti* Brocchi, 1876), previously considered to be extinct, is among the most emblematic [17]. Prior to 2003, the lizard species had been described from a single specimen (holotype by monotypy) collected around 1872 by the French botanist Benjamin Balansa at an unknown location in New Caledonia. The species was rediscovered by Ivan Ineich more than 130 years later, in December 2003, during an expedition devoted to the study of sea snakes on a small islet off the Isle of Pines. The specimen was captured by hand and later released after tissue samples and pictures were taken [22]. *P. bocourti* is remarkable because of its large size and pronounced dentition; its elongated, curved, and sharp teeth suggest it is a predator, an unusual trophic position since larger skink species are generally herbivorous and/or frugivorous. Its diet has been hypothesized to include larger invertebrates (crabs, mollusks, worms, insects, and spiders), other lizards, young birds, and different kinds of eggs [17,23,24]. Those teeth and its historical characters give that lizard its common name “Bocourt’s terrific skink” in relation with its generic name (*Phoboscincus*): “The name *Phoboscincus* combines the Greek noun for “fear” (*phobos*) with the Latin noun for “skink” (*scincus*) to give a name that should emphasize the awe-inspiring aspect imparted to these skinks by their large size and sharply pointed teeth” [25]. Indeed, its total length is about 50 cm, making it the second largest lizard species in New Caledonia (the first largest being the extinct goanna species mentioned above) and the largest predator lizard in the archipelago. 

In 2005, researchers returned to the islet where *P. bocourti* was rediscovered to study the species and obtain the first information on its natural history. At the request of the environmental authorities of the Southern Province of New Caledonia, a conservation plan was established that included several recommendations [24]. Since that time, no additional data on the biology, trophic interactions, and role of the species in the ecosystem have been obtained that would allow the established conservation plan to be followed. As a potential top predator, *P. bocourti* could have a significant impact on the island’s lower trophic levels (e.g. [1,24,26]), but more importantly, from a conservation point of view, it may also be very vulnerable to changes or disturbances in the ecosystem, thus making it a prime candidate for extinction. Such disturbances may include habitat fragmentation, tourism-related development, invasive species (rats and ants), environmental pollution, diseases, climate change, wildfire and prey availability [27]. Species vulnerability seems to be higher in general in island ecosystems; human-induced extinction rates are much higher on islands than on continents. Invasive species in particular are responsible for a large number of the documented extinctions of island vertebrates [28,29].

Basic biological information on *P. bocourti* is scarce; specifically, its population size, trophic interactions, and biological adaptations remain uncharacterized. Such data are essential to the identification of a species’ future risk of extinction and the establishment of clear conservation guidelines. Yet obtaining these data can be extremely challenging, particularly when population size is small or individuals are cryptic, making it difficult to capture or monitor the species [30,31]. Our study aims to elucidate the trophic ecology of *P. bocourti* using a multidisciplinary approach involving natural history, anatomy, morphology, genetics, and stable isotopes. In addition to the 2003 and 2005 expeditions, two additional trips were made to the islet in 2012 to collect more biological data on this elusive species. The goal was to observe the lizard in its natural habitat (feeding, reproduction, distribution, etc.), characterize potential threats to its well-being, and sample its tissues. We were able to amplify a small fragment of the mitochondrial 12S rDNA gene from the sole museum specimen of *P. bocourti*, the adult holotype collected around 1872 and deposited in the collection of Paris Natural History Museum (MNHN 3029). We compared the holotype’s sequence with those obtained from four individuals sampled during the 2005 and 2012 expeditions. We were also able to amplify larger fragments of the 12S rDNA and 16S rDNA genes from the four more recently sampled individuals, thus allowing us to examine genetic variability among them. We used x-ray microtomography to visualize the holotype’s skull and dentition. We employed stable isotopes to determine *P. bocourti*’s trophic position in the islet’s food web and thus test the hypothesis that it preys upon sympatric terrestrial vertebrates such as rodents, birds, and other reptiles. Because traditional trophic analysis techniques, e.g. stomach content sampling and stomach flushing, should not be used on endangered species, the development of indirect biochemical approaches such as analyzing the stable isotope ratios of animal tissues has been a boon in conservation studies. Finally, we discuss the importance of our different results in the context of the lizard’s rediscovery and the urgent need to establish clear conservation perspectives and priorities.

## Materials and Methods

### Field research on *P. bocourti* and its potential prey

**Figure 1:**
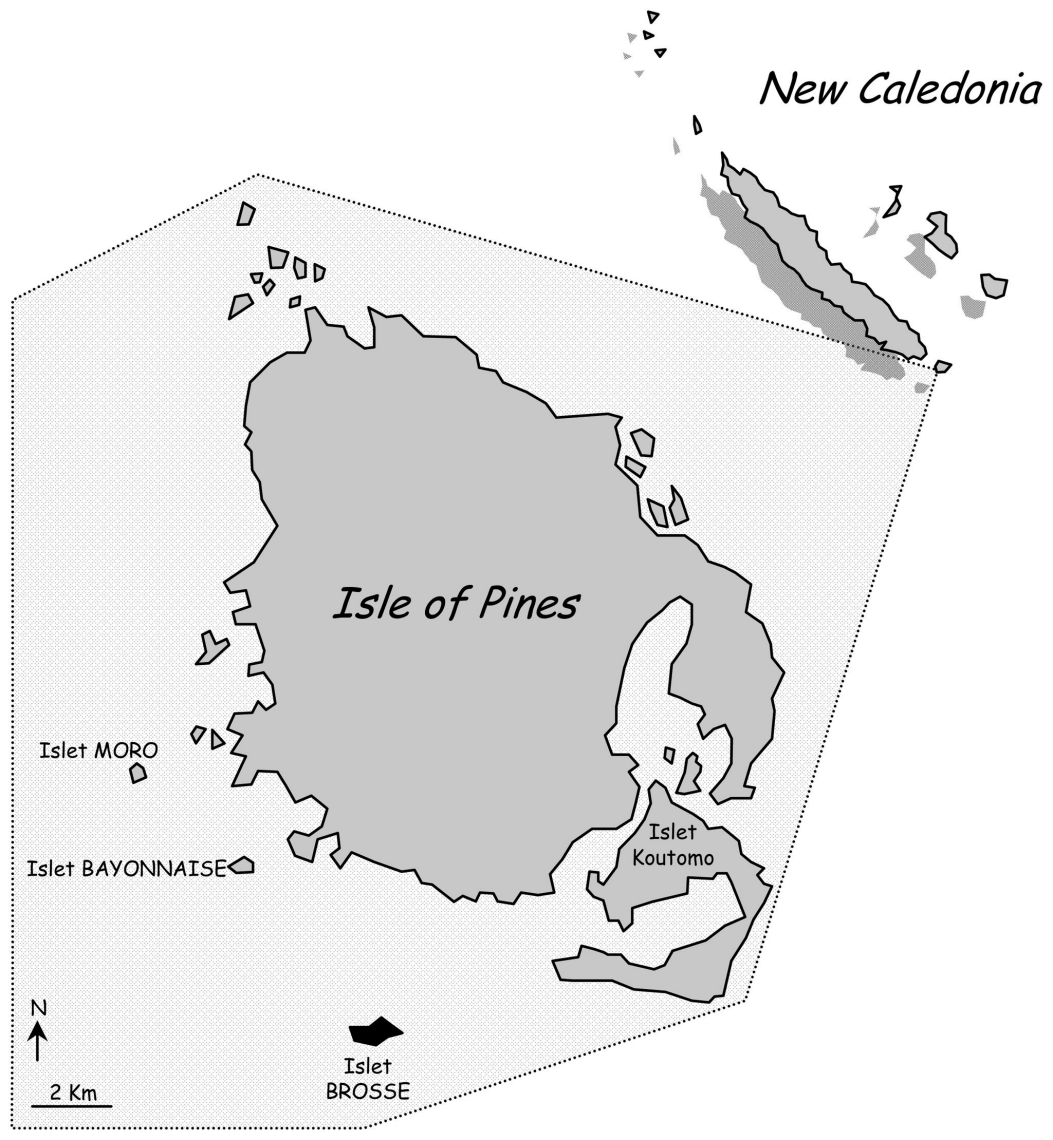
Map of Isle of Pines (French: Île des Pins) and Brosse Islet (in black), New Caledonia (Pacific Ocean).

Brosse Islet is a small, remote, coral island that is located to the southwest of the Isle of Pines at the southern end of New Caledonia (22°42'29"S, 167°27'38"E, Figure 1). It is southeast of Grande Terre, New Caledonia's main island, and 100 kilometers southeast of Nouméa, the capital. It is 60 ha in size (550 × 1400 m) and 9 m above sea level. It experiences four seasons that differ in temperature and rainfall; the hot and humid season from December to March, during which cyclones occur, and the cold and dry season from July to October are separated from each other by intermediate seasons [32]. Global positioning system (GPS) mapping revealed that the flat landscape is covered by plants such as ferns and *Pandanus* spp. The arboreal stratum is dominated by *Araucaria columnaris*, although sometimes other large trees like *Ficus* sp. are present.

Since the species is very difficult to observe and capture, our objective during the 2005 and 2012 field expeditions (14 days in Nov-Dec 2005, 19 days in Jan-Feb 2012, and 22 days in Nov-Dec 2012) was to observe the skink in its natural habitat and, if possible, live capture individual lizards so as to gain better knowledge of the species’ biology. We used a multitude of different and complementary approaches to meet this objective. First, in 2005, we performed visual surveys along a 400-m forest trail running from the sea to the center of the islet. The trail was completely cleared of leaves, branches, and rocks so that surveys could be conducted silently. Surveys took place at several points during the day (1 hour/day/person between 8 am and 6 pm) and at night (30 minutes/day/person between 6pm and 10pm). Second, in 2012, camera traps were used (one during the Jan-Feb trip and three during the Nov-Dec trip). They were placed in habitats identical to those in which *P. bocourti* had previously been observed and were programmed to take a picture every minute both day and night. Cameras were checked every two days. Third, in both 2005 and 2012, funnel traps were used and checked twice daily (6 traps/day in Nov 2005 and 2 traps/day in Jan-Feb 2012) (checked two times each day). Fourth, in 2005, pitfall trapping was conducted (5 traps/day); traps were checked twice a day. During all three expeditions, randomly directed visual surveys were performed (1 hour/day/person), as were observations from a hide placed at ground level (1 hour/day/person). Fruit, chicken eggs, lizards, as well as wet and dry dog and cat food were used as bait, although none of them seemed to be particularly efficient. All traps were placed in a random way inland in the forest on all parts of the islet. Lizards were collected or observed in all kind of habitats, however the islet vegetation is relatively homogeneous and the major differences are related only to the distance from the beach. 

Since we were uncertain to obtain direct information on the species’ diet through observation and/or the live capture of individual lizards, we decided in Jan-Feb of 2012 to use indirect methods to assess the trophic ecology of *P. bocourti*, namely the analysis of stable carbon and nitrogen isotope ratios. We therefore characterized the isotopic values of all potential food sources (at least those that were readily available on the islet) and compared them with those obtained from *P. bocourti*. Sampling of potential food sources took place as follows. Several individuals of all plant species (seeds or fruits) and mushrooms were collected by hand. Arthropods were captured with insect glue traps placed at ground level or on tree trunks or by hand during daily surveys of three hour duration each throughout the inner islet. Individuals were stored in ethanol and the whole body was used for isotopic analysis. Traps were placed in all types of habitat throughout the islet (20 traps/day in Nov-Dec 2005, 40 traps/day in Jan-Feb 2012, and 80 traps/day in Nov-Dec 2012) and checked three times a day. Reptiles were mostly caught by hand or occasionally by glue traps and a tail muscle sample were collected. Seabird samples consisted of muscle tissue from freshly-dead seabirds or abandoned eggs found in the ground. Rodents were captured using metallic traps and were sacrificed by cervical dislocation. Their muscle tissue was sampled for isotopic analysis. The traps were arranged in a circle with a radius of approximately 500 m that was centered in the southern part of the islet and largely encompassed the habitat in which the two *P. bocourti* had been observed in 2003 and 2005.

Because we did not have the permits to sample the entire ecosystem during each of the three most recent expeditions, we sampled the tail muscle of five different lizard species - *Bavaya cyclura, Bavayia sauvagii*, *Caledoniscincus austrocaledonicus*, *Caledoniscincus haplorhinus*, and *Lioscincus nigrofasciolatum* - in Nov-Dec 2005, Jan-Feb 2012, and Nov-Dec 2012 to estimate the variation in the isotopic baseline across those three trips. All biological samples were stored in 90% alcohol until they could be identified in the laboratory and analyses could be conducted. We used reptile and rodent muscle, bird feathers, and entire invertebrate corpses in our isotopic analyses. Most potential prey species were photographed and identified to the most useful classification level (species, genus, family, or order).

Animals in this study were treated humanely and in accordance with the laws of France and the French Overseas Territories (Decret 2003-768/NOR: AGRD0300394D). S. Caut was authorized by the French Minister of Agriculture (R-45GRETA-F1-04). Research protocols, licenses and permission to work, handle and collect were issued and ethical approved by the governments of France and New Caledonia (permits n° 62-2012/ARR/DENV of 9 January 2012 and n° 2806-2012/ARR/DENV of 6 December 2012).

### X-ray microtomography and 3D visualization

We used a Viscom X8050-16 μCT scanner located at the Center for Microtomography at the University of Poitiers (France) to scan the *P. bocourti* holotype. The x-ray source was a Viscom microfocus open tube (150kV) set to 90 kV and 0.6 mA. The detector was an image intensifier coupled with a camera; projections were 1004 x 1004 pixels with a pixel size of 147 µm. A voxel size of 74.7 µm was obtained in the reconstructed 3D images. Reconstruction was performed using ImageJ software (available from http://rsb.info.nih.gov/ij) and the FDK algorithms in DigiCT v.2.4.3 (Digisens, with the SnapCT plug-in - acceleration module on GPU). The rendering of the skull consisted of 1800 projections taken over 360°, with 4 integrations per projection. 3D images were produced in 16-bit voxels and subsequently converted into 8-bit voxels for visualization. Three-dimensional processing and rendering were obtained after semi-automatic segmentation of the cranial skeleton was performed using the functions ‘generate surface’ and ‘volume rendering’ in AVIZO 7.01 (VSG, SAS, Merignac, France, http://www.vsg3d.com).

### Isotopic analysis

There are several techniques that can be used to assess an organism’s diet, including traditional methods, such as foraging observations or stomach and feces analysis, or more recently developed approaches, such as the examination of stable isotope ratios in an animal’s tissues. In this study, direct observation was problematic because the species was rarely spotted and captured. Analysis of stomach contents using stomach flushing would also have been problematic because the procedure is invasive and can result in death, an unacceptable risk when dealing with an endangered species. Since the stomach and internal organs were completely removed from the holotype, probably at the time of collection, there was no possibility of analyzing its stomach contents. Moreover, observations of feeding behavior are frequently biased because it is often easier to see animals feeding on a certain type of prey or in a certain habitat. Results obtained from stomach or feces content analyses may also be skewed since they show the type and quantity of food a species has eaten recently, but cannot take into account differences in the retention times and digestibility of different dietary components [33,34]; for instance, food eaten by a prey species (or even its parasites) may be found in the predator’s stomach and thus wrongly be assumed to form a part of the predator’s diet. 

Samples from muscle of captured reptiles and samples from potential *P. bocourti* food items were collected for stable isotope analysis were dried at 60°C for 48 h, ground to a fine powder, weighed in tin capsules, and stored in a desiccator until analyses could take place. Stable isotope ratios of carbon and nitrogen in the samples were determined using an Optima® mass spectrometer (Micromass, UK) coupled to a C-N-S elemental analyser (Carlo Erba, Italy). Ratios are presented as δ values (‰), which represent the deviation of the sample from the vPDB (Vienna Peedee Belemnite) standard in the case of carbon and atmospheric N_2_ in the case of nitrogen. Stable C and N isotope ratios are calculated as follows: δ^13^C or δ^15^N= [(*R*_sample_/*R*_standard_)-1] x 1000, where *R* is ^13^C/^12^C or ^15^N/^14^N for δ^13^C or δ^15^N, respectively. Reference materials were IAEA-CH-6 (-10.4‰) for δ^13^C and IAEA-N1 (+ 0.4‰) for δ^15^N. One hundred replicate assays of internal laboratory standards indicate maximum measurement errors (SD) of ± 0.2‰ for carbon and ± 0.15‰ for nitrogen. C/N was the ratio of the total percentage of carbon to the total percentage of nitrogen.

Two sets of linear mixed models were evaluated. First, the isotopic baseline across time was examined using models in which the dependent variable was either the δ^13^C or δ^15^N value of individuals of the five reptile species consistently sampled (*B. cyclura, B. sauvagii*, *C. austrocaledonicus*, *C. haplorhinus*, and *L. nigrofasciolatum*), the independent variable was expedition date (Nov-Dec 2005, Jan-Feb 2012, and Nov-Dec 2012); reptile species was included as a random effect. Second, the difference in isotope ratios among all the reptile species was examined using models in which the dependent variable was either the δ^13^C or δ^15^N values and the independent variable was reptile species; expedition date was included as a random effect. We used Tukey’s HSD to test post-hoc differences between each pair of reptile species. The normality of the dependent variables was verified before models were fitted. Statistical analyses were performed using Statistica 6.0 (StatSoft Inc. 2001); the alpha level was P < 0.05.

### Molecular analyses

DNA from the MNHN holotype and the four recently sampled individuals of *P. bocourti* (one in Nov-Dec 2005 and three in Nov-Dec 2012) was extracted using the Chelex extraction process (Bio-Rad®, Hercules, 12 CA, U.S.A.; [35]) but employing a slightly modified protocol. Tail muscle tissue (2 mm^3^) was cut into small pieces, and samples were incubated for 2h at 57°C in 160 μl of 5% Chelex with 40 μl Proteinase K to increase tissue digestion. Following the incubation period, the samples were kept at 100°C for 15 minutes. After 4 min of centrifugation at 12500 rpm, the sample supernatants were transferred into 1.5 ml tubes. Negative controls were included in each extraction to monitor for contamination. In order to clarify the taxonomic relationship between the holotype and the more recently sampled lizards, we attempted to amplify a fragment of the mitochondrial 12S gene from all the samples using universal mitochondrial vertebrate primers [36]. Nevertheless, all attempts to amplify 12S rDNA from the holotype were unsuccessful because of contamination by human DNA. To overcome this problem, and the problem presented by the likely denaturation of DNA in the holotype, we used a previously designed short internal 12S rDNA forward primer to initiate amplification at universal markers: primer Lio12SF (5´- GTCGCCAGCTTACCTTGYAAAAGAA-3´ [37]) and universal 12SR (5´-GAGGGTGACGGGCGGTGTGT-3´ [36]). All PCR products were re-amplified because initial amplification was weak. Once this gene fragment had been successfully amplified from the holotype, we sequenced a longer 12S rDNA fragment as well as a 16S rDNA fragment from the four more recently sampled individuals to assess the species’ taxonomic relationship with other skinks. The following primers were used: 12S rDNA: 12SA 5´-AAACTGGGATTAGATACCCCACTAT-3´ and 12SB 5´-GAGGGTGACGGGCGGTGTGT-3´ [34] amplified a ~400 base pair fragment while 16S rDNA 5´-GCCTGTTTATCAAAAACAT-3´ and 16SH 5´-CCGGTCTGAACTCAGATCACGT- 3´ [38] amplified a ~700 base pair fragment. Excess primers and dNTPs were removed from the PCR product using an enzymatic reaction; *Escherichia coli* exonuclease I, Antartic phosphatase, and Antartic phosphatase buffer (all New England Biolabs) were added to each sample. Sequencing was carried out in both directions using the BigDye® Terminator v1.1 cycle sequencing kit (Applied Biosystems) in accordance with the manufacturer's instructions. Labeled fragments were resolved on an automated A3130xl genetic analyzer (Applied Biosystems). Incomplete terminal sequences and PCR primers were removed. 

Templates were sequenced on both strands, and the complementary reads were used to resolve rare, ambiguous base calls in Sequencher v.4.9. Sequences were aligned by eye in Seaview v.4.2.12 [39]. 

In order to assess the phylogenetic relationship between *P. bocourti* and other skinks, we performed Bayesian inference analyses using at least one species per genus for those genera included in Ineich et al. [40], which made 12S and 16S rDNA sequences available through Genbank. In addition, to root the phylogenetic tree, we employed three different genera known to be sister taxa to the New Caledonian skink group and that had been used in abovementioned study: *Morethia adelaidensis*, *Niveoscincus pretiosus*, and *Saproscincus mustelinus*. 

The most appropriate nucleotide substitution model was determined using the Bayesian Information Criterion (BIC) procedure in jModeltest v.0.1.1 [41]. The best-fitting model, TIM2+I+G, was employed in MrBayes to construct the tree using the Bayesian inference (BI) optimality criteria. The program’s default priors and Markov chain settings were used, and starting trees were random. Each run consisted of four chains of 10,000,000 generations that were sampled every 10,000 generations, resulting in a total of 750 trees. A plateau was reached after a few generations with 25% of the trees resulting from discarded burn-ins. 

## Results and Discussion

Four *P. bocourti* were captured over 55 days of intensive trapping on Brosse Islet (one in Nov 2005, none in Jan-Feb 2012, and three in Nov-Dec 2012). Another four were observed during visual surveys or on camera trap images (one in Jan-Feb 2012 and three in Nov-Dec 2012). Thus, even though one or two experienced field herpetologists spent 55 days trapping and surveying for this species, no more than 1 lizard per 6.875 days was observed or captured. In Nov-Dec of 2012, camera traps generated about 80,000 pictures over 22 days and nights of continuous field work and yet yielded only a few pictures of three distinct *P. bocourti* individuals. Five other most commonly terrestrial reptiles, *B. cyclura*, *C. haplorhinus*, *L. nigrofasciolatum*, *C. austrocaledonicus*, and *Rhacodactylus leachianus*, as well as one sea krait (*Laticauda saintgironsi*) were also sampled over the course of the different field expeditions.

### First Tracks of the Bocourt’s Terrific Skink Trophic Ecology

#### Contribution of teeth anatomy

The total length of *P. bocourti* makes it the largest native terrestrial squamate predator living in New Caledonia. However, little is known about its natural history, although its large size and enlarged teeth suggest that it may feed on other vertebrates [17,24]. It is unclear just how terrific Bocourt’s terrific skink is. Certainly, many scincomorph lizards opportunistically feed on insects, and some species demonstrate some degree of dietary specialization. The question is whether dentition is of taxonomic value or whether it simply represents an adaptation to a special diet in a monophyletic lineage. *P. bocourti* only shares its dentition with *P. garnieri*, the only other identified species of the genus, whose dental morphology certainly reflects its feeding habits. 

**Figure 2:**
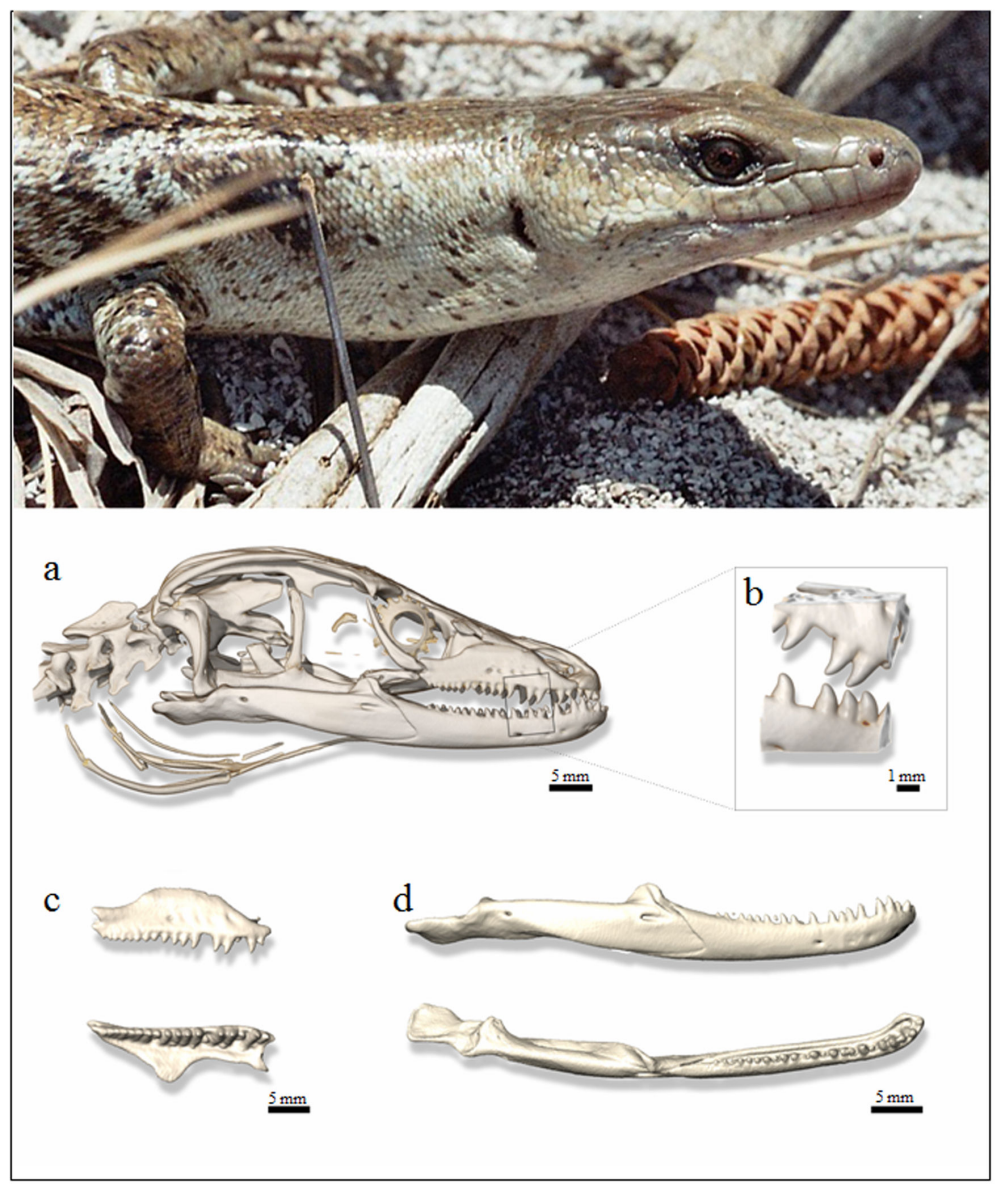
X-ray microtomography and 3D visualization. Top: anterior view of the body of the *Phoboscincus bocourti* captured during the November 2005 expedition to Brosse Islet. Bottom: volume rendering of the skull of the *P. bocourti* holotype - (a) Lateral view of skull including first vertebrae and hyoid apparatus; (b) Magnification of dentary and maxillary areas; (c) Morphology of dentary and maxillary teeth; (d) Lateral and dorsal view of dentary.

Since the feeding habits of *P. bocourti* are unknown, its dentition may help reveal its diet. The *P. bocourti* holotype has conical, sharply pointed teeth, and its anterior teeth are enlarged at the base and posteriorly curved. Teeth place were counted on head bones and are there indicated on both side, right/left. We used abbreviations DEN for dentary, PMX for premaxillary and MAX for maxillary: *P. bocourti*: DEN 23/23 ; PMX 11 ; MAX 18/19 (Figure 2). *P. bocourti* shows a lower number of teeth place on both sides on dentary and maxillary compared to its smaller congener *P. garnieri*, but curiously their premaxillary teeth place are identical in number (DEN 30/31 ; PMX 11 ; MAX 21/24). Teeth similarity between *P. bocourti* and *P. garnieri* certainly originated from common ancestry and not convergent evolution. However superficial similarity may not necessarily reflect common diet between both species. *P. bocourti*’s teeth are tightly spaced (their bases nearly touch); they lack enamel striations and have neither flattened nor sharpened surfaces at their tips. The species dentition can be described as heterodont and proterodont; the anterior teeth are 4-5 times larger than the posterior teeth on both the dentary and the maxillary. The anterior teeth resemble dog canines (rounded anterior faces) whereas the posterior teeth are more conical, with similar anterior and posterior slopes. According to Kosma’s classification of dental morphology [42], *P. bocourti* has a type A dental type, which is characterized by unicuspid recurved teeth. Such fang-like teeth are often seen in burrowing skinks and in the symphyseal region of the mandibles of many lacertids and teiids.

Pointed teeth are very useful in grasping and perforating the soft bodies of slugs, worms, or caterpillars. However, the teeth of *P. bocourti* are curved, and animals that feed on soft-bodied prey generally do not have curved teeth. Extremely pointed teeth are often associated with myrmecophagy; they also frequently lack enamel striations, which means they require less force to penetrate the cuticles of their arthropod prey: minute insects, especially termites and ants. The anterior teeth of *P. bocourti* are large, with wide, circular bases. While they are sharp, they are not sharply pointed, making them poorly adapted for a specialized diet of soft-bodied prey. Tooth elongation, which is not evident in *P. bocourti*, is often associated with minute or slippery prey items. Herbivory is common among the Scincomorpha and is often reflected by functional adaptations in tooth crown morphology that are clearly lacking in *P. bocourti*. In conclusion, our analysis of *P. bocourti*’s dentition suggests the species is neither a specialized vegetarian nor a specialist consumer of soft-bodied prey. It could prey on lizards since such prey are available year-round in New Caledonia. However, it is unlikely that *P. bocourti* is a lizard specialist, given the feeding habits of other Scincomorpha species. In addition to being saurophageous, the species most likely occasionally consumes invertebrates (insects, worms), bird eggs, and perhaps even small rats. It has yet to be demonstrated that *P. bocourti* eats plant matter.

#### Contribution of stable isotope analysis

Stable isotope analysis is especially advantageous when investigating the feeding ecology and habitat use of rare species of which direct observations are difficult to obtain [43,44]. Stable isotopes also provide time-integrated information on assimilated foods [45], which contrasts with the information generated by stomach analyses, in which only ingested foods are observed. As a result, stable isotopes are more and more commonly used to investigate diet composition and consumer trophic level [46-48]. Nitrogen (δ^15^N) and carbon (δ^13^C) stable isotope ratios are used, respectively, to reveal a species’ position within its food web and to trace the origin of the trophic resources it exploits [49]. 

**Figure 3:**
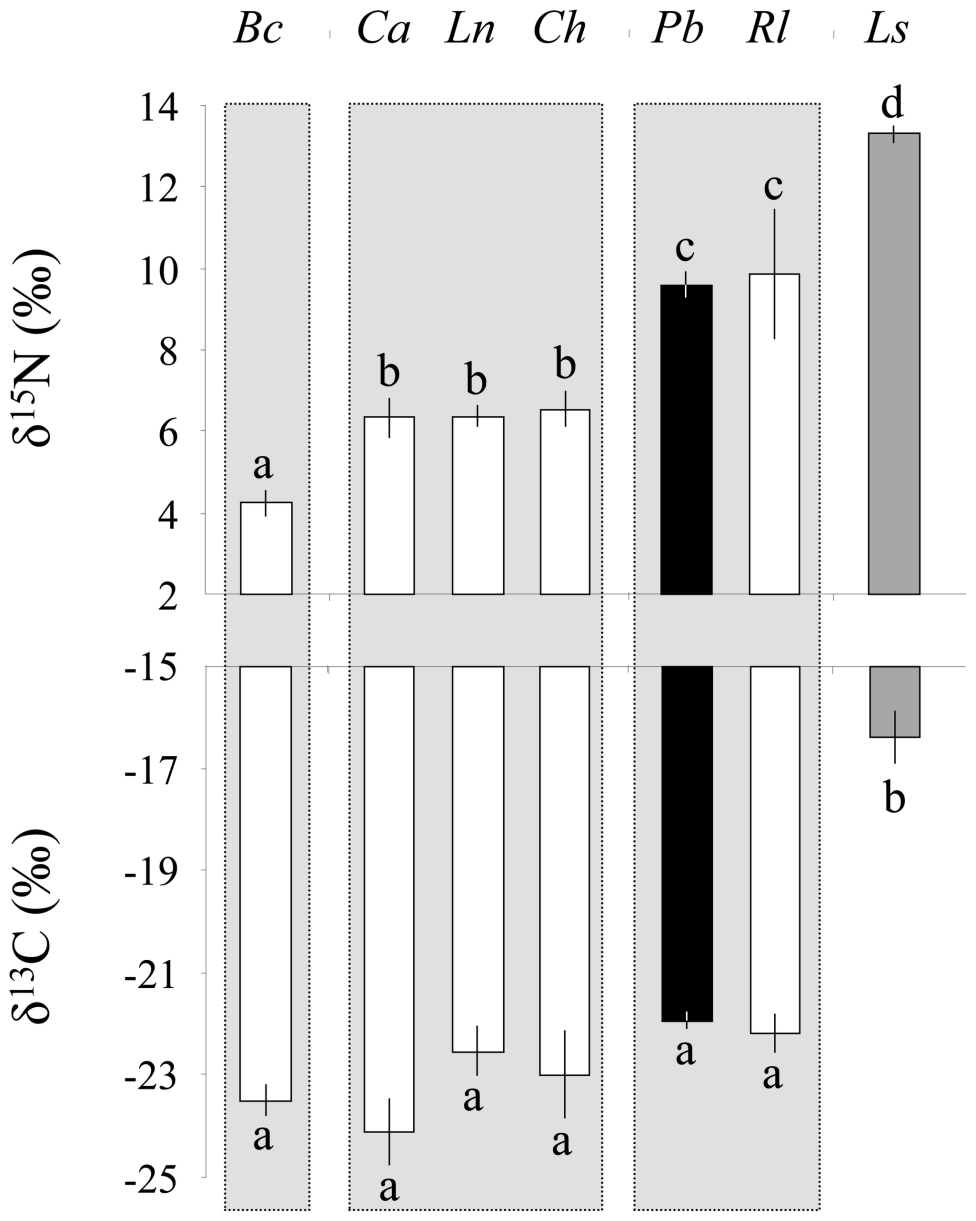
Variation in the δ^15^N and δ^13^C values (mean ± SE) of terrestrial reptiles. Abbreviations of lizard species are as follows: *Bavayia cyclura* (*Bc*), *Caledoniscincus haplorhinus* (*Ch*), *Lioscincus nigrofasciolatum* (*Ln*), *Caledoniscincus austrocaledonicus* (Ca), *Phoboscincus bocourti* (Pb), and *Rhacodactylus leachianus* (*Rl*). Samples were also obtained for the sea krait *Laticauda saintgironsi* (*Ls*). Different letters indicate significant differences in the isotopic signatures of the different species (P < 0.05) as determined using a Tukey’s HSD post-hoc test. Reptile species have been ordered by relative trophic nitrogen enrichment.

**Figure 4:**
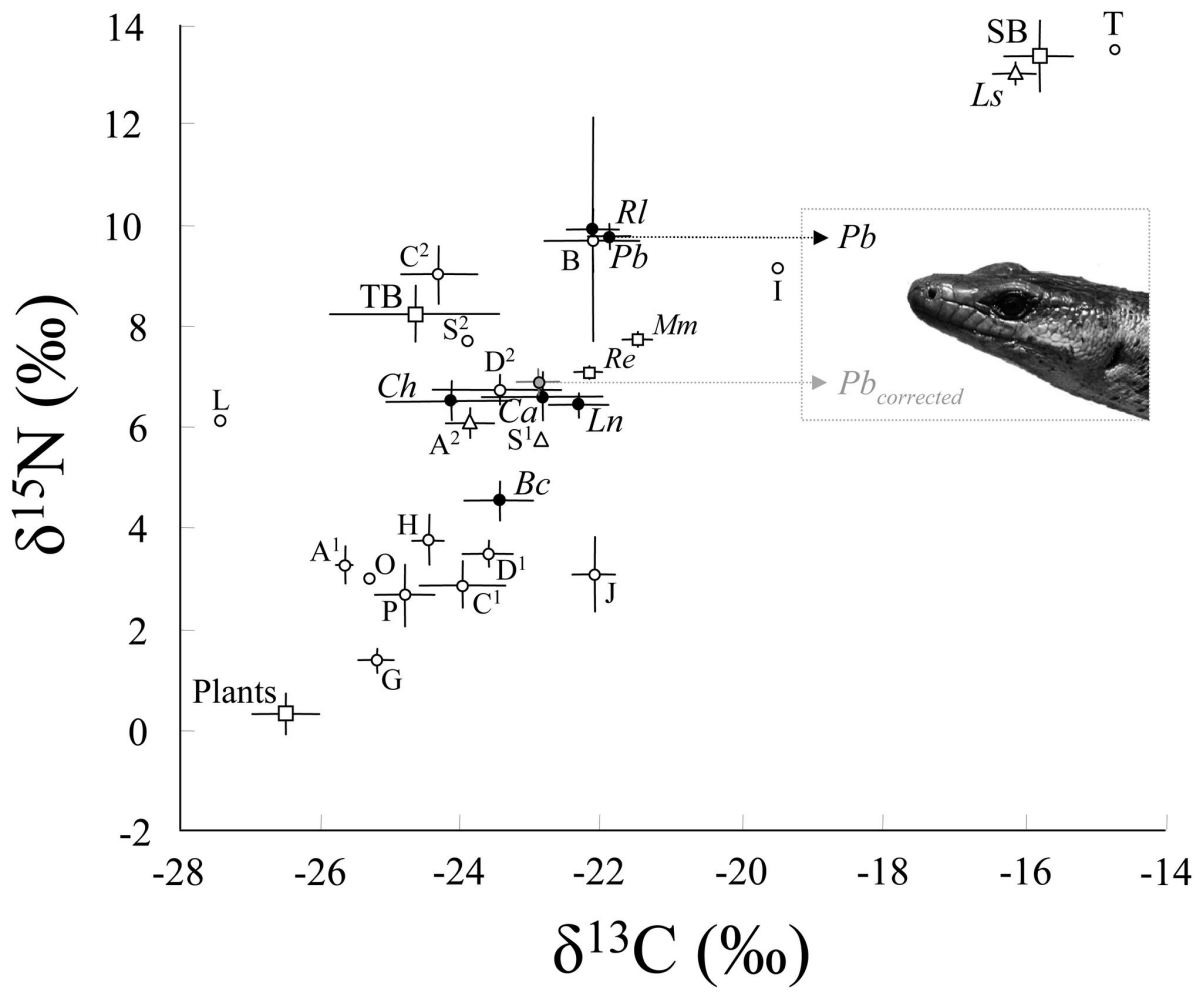
Mean ± SE of δ^15^N and δ^13^C values of animals and plants sampled on Brousse Islet. Plants are placed in a single group. Abbreviations for the terrestrial reptiles and sea krait are as follows: *Bavayia cyclura* (*Bc*), *Caledoniscincus haplorhinus* (*Ch*), *Lioscincus nigrofasciolatum* (*Ln*), *Caledoniscincus austrocaledonicus* (Ca), *Phoboscincus bocourti* (Pb), *Rhacodactylus leachianus* (*Rl*) and *Laticauda saintgironsi* (*Ls*). Invertebrates: Isopoda (I), Coleoptera (C^1^ and C^2^), Dyctioptera (D^1^), Diptera (D^2^), Julidae (J), Hymenoptera (H), Lepidoptera (L), Scolopendra (S^1^), Scorpiones (S^2^), Orthoptera (O), Annelida (A^1^), Araneae (A^2^), Gastropoda (G), Paguridae (P), Brachyura (B), and seakrait tick (T). Rodents: *Rattus exulens* (Re) and *Mus musculus* (Mm). Birds are grouped as terrestrial birds (TB) and seabirds (SB). The isotopic values of terrestrial reptiles are represented by black, filled points. The grey point is the mean discrimination-corrected isotopic value of *P. bocourti* (Pb_corrected_), which is superimposed onto its prey to visually illustrate its possible diet and trophic relationships. Fractionation values are 1‰ for δ^13^C and 3‰ for δ^15^N.

As we collected samples from predators and prey over the three research expeditions, we were able to verify that the isotopic baselines of carbon and nitrogen did not differ among trips (δ^13^C: F_2,26_ = 2.71, P = 0.086, and δ^15^N: F_2,26_ = 1.25, P = 0.304). As a result, we were comfortable comparing the trophic positions of the different reptile species found on the islet; we observed a significant difference in their δ^15^N values (δ^15^N: F_6,31_ = 32.74, P < 0.001, Figure 3). Three distinct trophic groups were present, with *P. bocourti* and *R. leachianus* occurring at the top of terrestrial trophic web (Tukey’s HSD, P < 0.05, Figure 3). Reptile species did not differ in their carbon isotope ratios (δ^13^C: F_6,31_ = 11.54, P < 0.001, Figure 3), probably because producers vary little in their carbon isotope ratios (e.g. only C_3_ plants are present, Figure 4). In contrast, the sole marine reptile sampled in our study, the sea krait (*L. saintgironsi*), showed significant higher carbon and nitrogen isotope ratios, which confirms that it is an eel specialist [22].

Recently, different isotopic models have been used to reconstruct diets using the isotopic ratios observed in animal tissues (e.g. review [43,50]). However, quantitative mixing models have not been used because of their poor resolution of the geometry of the resource mixing space and the relatively large number of potential prey. Moreover, because δ^13^C and δ^15^N discrimination factors (i.e. the difference between the isotopic signature of the consumer and that of its prey) remain relative uncertain for terrestrial reptiles (and especially *P. bocourti*), mixing models will not lead to conclusive results if used to determine the diet composition [43,51-53]. To date, only one study has estimated this parameter in skinks and only for carbon [54,55]. However, although discrimination factors vary among tissues, taxa, and diets, their values are often situated between 0–1% for δ^13^C and 3–4% for δ^15^N [51]. Thus, when we compared *P. bocourti*’s isotopic signature (corrected for the discrimination factor) with those of all the potential prey available in the ecosystem (Figure 4), we found two important results. First, marine resources seem to be inexistent in the skink’s diet, even though it had been hypothesized that the species consumed seabird eggs or chicks [17,24]. Indeed, seabirds and sea kraits had much higher isotopic values than *P. bocourti*, and seabird-derived nutrients do not seem to enrich the islet’s ecosystem, in contrast to what has been observed on islands with large populations of seabirds (Figure 4, [56]). Second, our results show that *P. bocourti* seems to be a predator of terrestrial reptiles. We observed that its discrimination-corrected isotopic signature fits exactly with the isotopic signature of the middle trophic group of reptiles, which comprises *C. austrocaledonicus*, *C. haplorhinus*, and *L. nigrofasciolatum* (Figure 4). Our results are supported by the known diet of arboreal *R. leachianus*, which is one of the largest extant gekkotan lizards (maximum adult body length of 255 mm). *R. leachianus* occupies the same trophic position (δ^15^N) as *P. bocourti* and is known to consume smaller lizards, including conspecifics [17]. 

When we compared the isotopic values of the two other reptile groups (Figure 3) with results from traditional diet analyses obtained from the literature, they corresponded well. The arboreal gecko *B. cyclura* (max adult body length of 72 mm) occupies a lower trophic level and eats a variety of small arthropods. The two small skink species *C. austrocaledonicus* (max adult body length of 57 mm) and *C. haplorhinus* (max adult body length of 55 mm) occur at an intermediary trophic level and feed on a range of invertebrates, including spiders, crickets, amphipods, and Lepidoptera ([17], Figure 4). *L. nigrofasciolatum* (max adult body length of 112 mm) occupies the same trophic level and also consumes invertebrates, but additionally sporadically feeds on *Bavaya* spp. [57].

### The return of Bocourt’s terrific skink?

#### View from a genetic perspective

Increasing habitat destruction and the subsequent loss of biodiversity make it more and more important to preserve specimens in museum collections, especially when the populations and species from whence they came are extinct. In the future, such specimens may represent the sole source of molecular data for that species, a fact that may take on particular importance when species are rediscovered (43.6% of rediscoveries represent the first record of the species since its original description [12]). Indeed, the first step following rediscovery should be to compare the DNA of the “rediscovered” species with that of the original holotype to confirm the veracity of the claim, even if this step is not generally performed nor is it always possible. Indeed, the morphology of threatened and endangered species is most often assessed in the field since individuals are released after capture; however, this process can be difficult and lead to serious errors [58]. Unfortunately, most specimens collected before 1950 were fixed in formaldehyde, limiting their utility in DNA studies, even if they were later transferred to alcohol by a curator. Although several methods for extracting DNA from formaldehyde-fixed animal tissue have been developed, they require large amounts of tissue, which are rarely available, or recover only short fragments of DNA [59]. The findings obtained from our molecular analyses show that the *P. bocourti* holotype and the four more recently caught individuals are conspecifics. When their 12S rDNA gene fragments (141 base pairs) were aligned, their sequences were found to be identical. The 12S and 16S rDNA fragments (418 bp and 456 bp, respectively) sequenced for the four recently captured individuals showed a lack of genetic variation.

#### Rediscovery vs conservation

Although species rediscoveries are often celebrated by the media and help identify natural areas that are worth protecting, the real challenge is using rediscoveries to encourage national and local authorities to support future conservation efforts [60]. Indeed, the long-term survival of a rediscovered species cannot be guaranteed. A recent review by Scheffers et al. [12] suggests that the majority of rediscovered species remain acutely threatened; many species rediscovered decades ago are still classified as critically endangered, endangered, or vulnerable [2]. For example, the central rock-rat, *Zyzomys pedunculatus*, was rediscovered several times after assumed periods of extinction [61]. The greater akialoa, *Hemignathus ellisianus*, and the mythical ivory-billed woodpecker, *Campephilus principalis*, were rediscovered in 1969 [62] and 2004 [63], respectively, but neither has been observed since. Scheffers et al. [12] concluded that many of the 351 rediscovered species discussed in their study are likely to go extinct without significant conservation efforts. Since its rediscovery in 2003, neither a management plan nor protective measures have been implemented locally or internationally for *P. bocourti*, despite the official recommendations made in 2005 by I. Ineich. Despite our intensive surveys, we observed only 8 individuals of a very large lizard species on a very small island. Although this low observation rate could be explained by an unknown particularity of *P. bocourti*’s natural history (e.g. it lives in burrows or high in trees), it seems very likely that the size of the population must be small. However, given that a subadult was captured in 2012, there may be hope for its reproductive capacity, even if we still know nothing about this aspect of its biology. Our results regarding *P. bocourti*’s dentition and trophic ecology strongly suggest that it is a top predator in its ecosystem and a major consumer of small terrestrial reptiles. However, this position at the top of the food web is not a guarantee of survival. 

Indeed, one of the major conservation challenges identified during our field expeditions to Brosse Islet was the presence of introduced rodents: the Pacific rat (*Rattus exulans*) and the common mouse (*Mus musculus*). Rats are now found on 80% of the world’s islands and are major predators of birds, reptiles, and arthropods [29]. They are omnivorous and eat the seeds, stems, fruits, and leaves of many plant species [64-66]. Although they are not considered to constitute a threat to plant species, they are thought to have a greater global impact than carnivorous predators because they feed on both animal and vegetal resources, which may allow them to survive in relatively large numbers even when one resource type is temporally or spatially depleted. As a result, their trophic interactions with *P. bocourti* could be multifaceted; they may be direct if invasive rodents prey upon *P. bocourti* eggs or juveniles and/or indirect if rodents compete with the skinks for resources such as small lizards or invertebrates. Indeed, the stable isotope analysis shows that rodents occupy an intermediate level in the trophic web, occurring between *P. bocourti* and other terrestrial reptiles (Figure 4). Rodents may well compete with *P. bocourti* for small terrestrial reptiles (even if these prey represent only a small part of the rodent diet, Figure 4) or compete with the small terrestrial reptiles themselves (the mid-range reptile group) by consuming large quantities of invertebrates (as reflected by their isotopic values, Figure 4). All these interactions could negatively affect small reptile populations and, via trophic cascades, top predators such as *P. bocourti*. Moreover, even if we did not quantify it directly, we observed a worrying increase in rodent and anthropogenic (e.g. tourism) impacts between 2003 and 2012. Because invasive rodents are known to have substantial effects on natural ecosystems [67], their management or complete eradication is often a conservation priority [29,68,69]. There is thus an urgent need to seriously evaluate the possibility of their eradication on this islet, a process that requires a better knowledge of the rodents’ feeding biology [68].

It is highly probable that *P. bocourti* is also present on other islands of the archipelago, particularly those located near Brosse Islet, as suggested by Ineich [24]. Indeed, the holotype was probably collected on the eastern coast of Grande Terre or the Lifou Island in the Loyalty Islands (although the latter hypothesis seems less likely given the species composition of the Loyalty Islands which have distinct biogeographic origins) because Benjamin Balansa never visited the Isle of Pines and its small islets [24]. Recently, there has been increasing interest in the patterns of species rediscoveries [8,9], and two major opposing hypotheses have been proposed. The range collapse hypothesis suggests that species will be rediscovered at the center of their historical geographical ranges because of anthropogenic threats such as predation or habitat destruction along peripheries [70,71]). The range eclipse hypothesis suggests that rediscovery will take place at the peripheries of historical ranges because species have taken refuge in more secure peripheral habitats as a result of anthropogenic threats at the center [70,72]. A third, more recent hypothesis, the elevation refuge hypothesis, considers that elevation is an important geographical factor in species range contractions [73].

**Figure 5:**
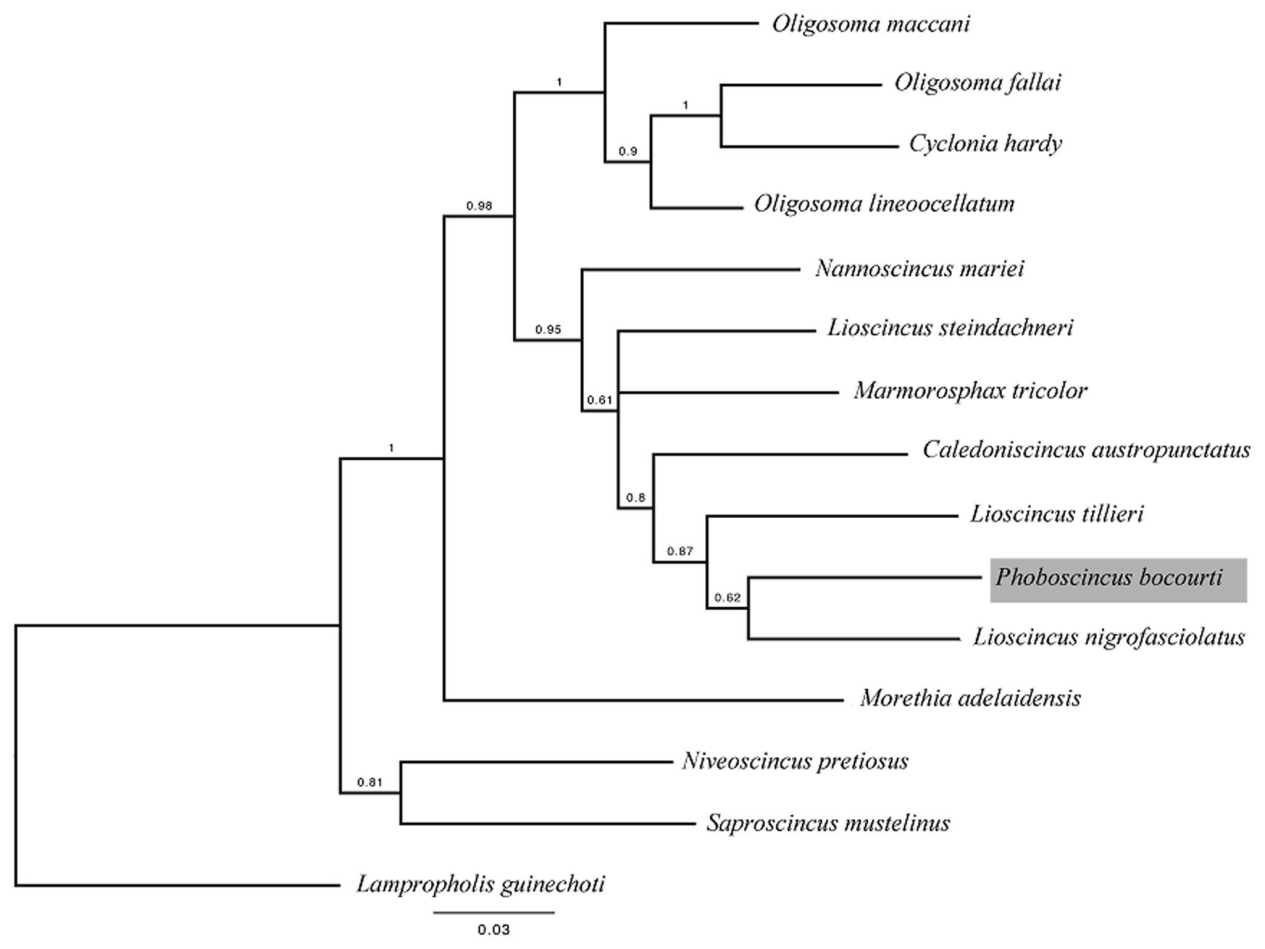
Bayesian Inference tree of the scincid species sampled based on partial sequencing of the 12S and 16S rDNA genes. The phylogram was obtained using Bayesian inference. Node values are the posterior probabilities recovered from the Bayesian inference analysis. The best-fitting model inferred from the alignment of 12S and 16S rDNA gene fragments (423 bp and 467 bp respectively, both fragments concatenated 887 bp, Genbank accessions: KF589953‏-45) for the BI tree was TIM2+I+G (−lnL=−3812.3087, BIC=7868.9798). In the phylogram, a poorly-resolved monophyletic clade (BPP: 0.62) was recovered, in which *P. bocourti* and *Lioscincus nigrofasciolatus* are sister clades. There was better support for *Lioscincus tillieri* being a sister group to the clade containing *P. bocourti* and *L. nigrofasciolatus* (BPP: 0.87). Our results confirm those of Ineich et al. (2013); some genera are paraphylectic (e.g. *Oligosoma* and *Lioscincus*), suggesting the need for taxonomic reassessment.

Our phylogenetic analyses corroborate some of Ineich et al.’s findings [40]: the clade containing the two species, *P. bocourti* and *L. nigrofasciolatum*, is monophyletic and these two are sister species (Figure 5). However, *P. garnieri* was not an additional sister species in our study, perhaps because the genes employed (NADH, ND2, and RAG1) by Ineich et al. differed from those we employed (12S rDNA and 16S rDNA). Furthermore, the high degree of genetic similarity to *L. nigrofasciolatum*, which has a widespread distribution in New Caledonia, suggests that genetic isolation has taken place at the geographical remote islet, where recent dispersal or colonization is highly improbable. The lack of genetic variation among the recently captured individuals may imply that a single lineage is present on the island, with few inter-island exchanges having occurred; however, further sampling is needed to test this hypothesis. Moreover, the degree of genetic divergence in the mitochondrial sequences (rates in Squamata are 0.45% and 0.5% per million years for 16S and 12S, respectively [74,75]) suggests that *P. bocourti* diverged from main island *L. nigrofasciolatum* around the Middle Miocene (Figure 5). All of these results are in accordance with the possibility that *P. bocourti* could be present in numerous other areas of New Caledonia, which may be more or less affected by anthropomorphic disturbances (e.g. invasive rodents and tourism).

It has been shown that climate change has been the cause of 4% of local lizard extinctions worldwide; by 2080, it is estimated that this percentage will reach 39% [76]. The identification of population declines is an important step in any conservation program [7]. Our study is the first to investigate *P. bocourti*’s diet, its place in the food web, and the extent of genetic variation in the only known extant population. *P. bocourti* is the largest terrestrial squamate predator alive in New Caledonia and probably plays a key role in the ecosystem, particularly on small islets like Brosse. It appears to function as a top predator and is thus likely to be very sensitive to habitat modifications, especially those caused by humans. We suggest that direct and indirect interactions with invasive rodents could pose a major threat to the survival of this species. We also underscore the urgent necessity to better characterize these potential competitors and quickly develop a management plan that will counter their effects on this threatened lizard species. Further biological information (e.g. genetic and ecological) is necessary to construct and implement a conservation program since no concrete measures have been taken since the rediscovery of Bocourt’s terrific skink almost 10 years ago.
